# Analysis of Stage-Specific Gene Expression Profiles in the Uterine Endometrium during Pregnancy in Pigs

**DOI:** 10.1371/journal.pone.0143436

**Published:** 2015-11-18

**Authors:** Mingoo Kim, Heewon Seo, Yohan Choi, Inkyu Yoo, Minseok Seo, Chang-Kyu Lee, Heebal Kim, Hakhyun Ka

**Affiliations:** 1 Division of Biological Science and Technology, Yonsei University, Wonju, Republic of Korea; 2 Interdisciplinary Program in Bioinformatics, Seoul National University, Seoul, Republic of Korea; 3 Department of Animal Biotechnology, Seoul National University, Seoul, Republic of Korea; 4 C&K Genomics, SNU Research Park, Seoul, Republic of Korea; University of Quebec at Trois-Rivieres, CANADA

## Abstract

The uterine endometrium plays a critical role in regulating the estrous cycle and the establishment and maintenance of pregnancy in mammalian species. Many studies have investigated the expression and function of genes in the uterine endometrium, but the global expression pattern of genes and relationships among genes differentially expressed in the uterine endometrium during gestation in pigs remain unclear. Thus, this study investigated global gene expression profiles using microarray in pigs. Diverse transcriptome analyses including clustering, network, and differentially expressed gene (DEG) analyses were performed to detect endometrial gene expression changes during the different gestation stages. In total, 6,991 genes were found to be differentially expressed by comparing genes expressed on day (D) 12 of pregnancy with those on D15, D30, D60, D90 and D114 of pregnancy, and clustering analysis of detected DEGs distinguished 8 clusters. Furthermore, several pregnancy-related hub genes such as *ALPPL2*, *RANBP17*, *NF1B*, *SPP1*, and *CST6* were discovered through network analysis. Finally, detected hub genes were technically validated by quantitative RT-PCR. These results suggest the complex network characteristics involved in uterine endometrial gene expression during pregnancy and indicate that diverse patterns of stage-specific gene expression and network connections may play a critical role in endometrial remodeling and in placental and fetal development to establish and maintenance of pregnancy in pigs.

## Introduction

The uterus plays a critical role in the control of the estrous cycle and pregnancy in pigs. During pregnancy, the uterus communicates with the conceptus (embryo/fetus and associated extraembryonic membranes) to establish and maintain pregnancy and it undergoes dramatic functional and morphological changes [1]. Synchronization of the developing embryo and appropriate endometrial remodeling is essential to a successful pregnancy, while failure leads to embryonic mortality. Accordingly, knowledge regarding the pattern of expression of uterine endometrial genes and their function in endometrial remodeling, embryo development and placentation during pregnancy is an important aspect of management of successful pregnancies. Thus, numerous studies have focused on elucidating the expression and function of uterine genes during pregnancy.

In pigs, maternal recognition of pregnancy, the extension of the functional lifespan of the corpora lutea, occurs on day (D) 12 of pregnancy, followed by embryo implantation, which continues until D18 of pregnancy [2, 3]. Once implantation is accomplished, the uterus participates in the formation of a maternal component of a true epitheliochorial type placenta during mid-to-late pregnancy, which transports nutrients to the developing embryos and exchanges gases. During this period placentation is completed, and rapid organogenesis of the developing fetuses occurs [4, 5]. In the late stage of pregnancy near term, the uterus experiences drastic changes in the expression of many genes and gene product function resulting from altered placental hormone secretion in order to prepare for parturition. For example, progesterone levels decrease, whereas estrogen levels increase [6]. These hormonal changes promote uterine contractility through their effects on myometrial contractile proteins, gap junction formation and increasing responsiveness of the uterus to oxytocin and prostaglandin (PG) F_2α_ [7].

Uterine endometrial gene expression during pregnancy is regulated mainly by steroid hormones such as progesterone and estrogen and cytokines from the ovaries and/or placenta [2, 8]. Gene products expressed in the endometrium in response to those hormones and cytokines include transport proteins (uteroferrin, retinol-binding protein and folate-binding protein), growth factors (fibroblast growth factor 7, insulin-like growth factor 1, epidermal growth factors, transforming growth factor-βs and connective tissue growth factor), enzymes (antileukoproteinase, cathepsins, lysozyme and β-hexoseaminidase), extracellular matrix proteins (osteopontin, fibronectin and vitronectin) and cell adhesion molecules (integrins α4, α5, αv, β1 and β3) [9–14]. These molecules are involved in the process of embryo implantation, membranogenesis, placentation, organogenesis and endometrial remodeling.

To investigate the pattern of uterine endometrial gene expression and gene function during pregnancy, a one-by-one approach was used before the genomic era. Although this approach has significantly helped us to understand gene expression and function in the uterus, a genome-wide approach using a microarray technique allows us to more efficiently investigate global gene expression in the uterus during different stages of pregnancy or in different physiological or pathological conditions. Many studies have applied microarray analysis to investigate expression of uterine endometrial genes during various stages of development or pregnancy and under pathological conditions. For example, gene expression profiles in the uterine endometrium during the implantation period have been analyzed using a genome-wide microarray technique in humans, mice and cows [15–17]. In pigs, microarray-based experiments have also been carried out to analyze differentially expressed genes in the endometrium during the implantation stage due to early exposure to estrogen [18] or during the estrous cycle [19]. However, there have been no previous reports on the global patterns of expression of genes and the relationships among genes affecting expression and function in the uterine endometrium during pregnancy. Therefore, in this study we analyzed global gene expression profiles using a microarray technique and performed three types of analyses: (1) detection of differentially expressed genes (DEGs) by comparing genes expressed in the uterine endometrium on D12 of pregnancy with those on D15, D30, D60, D90 and D114 of pregnancy; (2) clustering analysis to group those DEGs based on expression patterns during pregnancy; and (3) network analysis to find hub genes that are correlated with expression of other genes. From these analyses, we sought to identify novel uterine marker genes at different pregnancy stages, and to provide valuable insight into the molecular mechanisms regulating dynamic expression of uterine endometrial genes during pregnancy.

## Materials and Methods

### Animals and tissue preparation

All experimental procedures involving animals were conducted in accordance with the Guide for Care and Use of Research Animals in Teaching and Research and were approved by the Institutional Animal Care and Use Committee of Yonsei University (Approval No. YWC-P120). Eighteen Landrace and Yorkshire crossbred sexually mature gilts of similar age (8–10 months) were artificially inseminated at the onset of estrus (D0) and 24 h later with fresh boar semen and assigned to D12, D15, D30, D60, D90, or D114 pregnancy groups (n = 3 pigs per day). The reproductive tracts of gilts were obtained immediately after they were slaughtered at a local slaughterhouse and uterine endometrial tissues were harvested immediately. Pregnancy was confirmed by the presence of apparently normal filamentous conceptuses in uterine flushings on D12 and D15 and the presence of embryos and placenta at later dates. Uterine flushings were obtained by introducing and recovering 50 ml of phosphate buffered saline (PBS) (pH 7.4) after hysterectomy (25 ml/uterine horn). Endometrial tissues were dissected from the myometrium and placental tissues, collected from four different areas of the middle portion of the uterine horn, and mixed for analysis to reduce the heterogeneity of gene expression. Endometrial tissues on D30 to D114 were collected from where placental tissues were removed. Endometrial tissues were snap-frozen in liquid nitrogen and stored at -80°C for RNA extraction.

### Gene expression profiling

#### Total RNA extraction

Total RNA was extracted from endometrial tissues using TRIzol reagent (Invitrogen, Carlsbad, CA) according to the manufacturer’s recommendations. The quality of total RNA was evaluated using NanoDrop (NanoDrop Technologies, Wilmington, DE) and Experion (BioRad, Hercules, CA).

#### Target preparation, microarray hybridization and scanning

The GeneChip Porcine Genome Array (Affymetrix, Santa Clara, CA) used in this study contained 24,123 probe sets outlining 19,675 transcripts, which represented 11,265 genes [20]. Five micrograms of total RNA from porcine endometria were used for labeling. Probe synthesis from total RNA samples, hybridization, detection, and scanning were performed according to standard protocols from Affymetrix at Seoulin Bioscience Molecular Biology Center (Seoul, Korea). Briefly, cDNA was synthesized from total RNA using the One-Cycle cDNA Synthesis Kit (Affymetrix). Single stranded cDNA was synthesized using Superscript II reverse transcriptase and T7-oligo (dT) primers at 42°C for 1 h. Double stranded cDNA was obtained by using DNA ligase, DNA polymerase I and RNase H at 16°C for 2 h, and followed by T4 DNA polymerase at 16°C for 5 min for gap filling. After clean up with the Sample Cleanup Module (Affymetrix), double-strand cDNA was used for in vitro transcription (IVT). cDNA was transcribed using the GeneChip IVT Labeling Kit (Affymetrix) in the presence of biotin-labeled CTP and UTP. After clean up with the Sample Cleanup Module (Affymetrix), 10–15 μg of labeled cRNA was fragmented from 35 to 200 bp by fragmentation buffer (Affymetrix). Fragmented cRNA was hybridized to the porcine genome microarray chips (Affymetrix) at 45°C for 16 h according to the Affymetrix standard protocol. After hybridization, the arrays were washed in a GeneChip Fluidics Station 450 with a non-stringent wash buffer at 25°C followed by a stringent wash buffer at 50°C. After washing, the arrays were stained with a streptavidin-phycoerythrin complex. After staining, intensities were determined with the GeneChip scanner 3000 (Affymetrix) controlled by Gene Chip Operating System (GCOS) Affymetrix software.

### Data analysis

#### Differentially expressed genes (DEGs)

The quality of the array image was assessed as described in the Affymetrix GeneChip expression analysis manual. A robust multi-array averaging procedure (RMA) was implemented in the R statistical package [21]. Expression values were computed from the raw CEL files by applying the RMA model of probe-specific correction for perfect-match probes. The corrected probe values were then normalized via quantile normalization, and a median polish was applied to compute one expression measure from all probe values using the RMA package. Whole expressions were log_2_-transformed after normalized values were calculated by RMA and quantile normalization. The expression of individual genes on D12 to D114 was compared using the linear models for microarray data (LIMMA) analysis [22]. The Benjamini–Hochberg correction for false discovery rate (FDR) [23] was used for all probe-level normalized data. We defined genes as differentially expressed only if they met the criteria of FDR adjusted *P*-value < 0.05 in the unpaired Welch t-test. In addition, the DEGs were classified as either up- or down-regulated genes depending on fold change (+ or -, respectively) by calculating log_2_(gene expression level of later stage/gene expression level of earlier stage).

#### Clustering analysis

Soft clustering data were obtained using the Mfuzz package implemented in R [24, 25]. The raw ratios for the time profiles of DEG were log_10_ transformed and then normalized such that, for each profile, the mean was zero and the standard deviation was one. The transformed profiles were then clustered using the Mfuzz package. We used the fuzzy c-means (FCM) clustering algorithm, which is a part of the package. FCM clustering is a soft partitioning clustering method that requires two main parameters (*c* = number of clusters, *m* = fuzzification parameter) and uses Euclidean distance as the distance metric. FCM assigns to each profile a membership value in the range (0, 1) for each *c* cluster. The algorithm iteratively assigns the profile to the cluster with the nearest cluster center while minimizing an objective function. Parameter *m* plays an important role in deriving robust clusters that are not greatly influenced by noise and random artifacts in data. For our analysis, the optimal values of *c* and *m* were derived by the iterative refinement procedure as previously described [26]. The final clustering was done with parameters *c* = 8 and *m* = 1.25.

#### Functional annotation clustering analysis using the DAVID tool

For investigating gene-set level patterns, we employed the DAVID functional annotation tool [27]. Annotated porcine gene information for DEG probe identifications was obtained from NetAffx (http://www.affymetrix.com/analysis/index.affx). Because of the limited number of annotated genes for the porcine genome in NetAffx resulting from the limited availability of the full-length porcine cDNA sequence, we used human gene symbols annotated for the Affymetrix porcine genome microarray probe identifications for the genes whose annotations were not available in NetAffx, as described in Tsai et al. [20]. Functional annotation clustering analysis using porcine and human gene symbols in the DAVID program was conducted at the highest stringency to identify the biological function of DEGs in the uterine endometrium during pregnancy. Annotation clusters having a low enrichment score (< 1.3) were filtered out because most of the included biological terms were not significant (*P*-value < 0.05).

#### Weighted gene co-expression network analysis (WGCNA) and module construction

Module detection and characterization were performed using customized R software functions. The absolute value of the Pearson correlation between expression profiles of all pairs of genes was determined. Then, the Pearson correlation measure was transformed into a connection strength measure by using a power function ${Edge}_{ij}\;={\left|r_{ij}^{2}\right|}^{\beta }$ [28]. The connectivity measure for each gene is the sum of the connection strengths (correlation β) between the gene and all the other genes in the network. Gene expression networks, like virtually all types of biological networks, exhibit an approximate scale-free topology. The coefficient of determination between log p(k) and log(k) was used to determine how well a resulting network fit the scale-free topology for a range of values. The scale-free topology criterion [28] was used to determine the power. To group genes with coherent expression profiles into modules, we used average linkage hierarchical clustering, which uses the topological overlap measure as dissimilarity. The topological overlap of two nodes reflects their similarity in terms of the commonality of the nodes they connect to each other [29, 30]. A height cutoff value of 0.94 was chosen and obtained 5 gene co-expression modules in this study.

### Quantitative real-time RT-PCR

To technically validate the detected results from microarray analysis such as clustering, network, and DEG finding analysis in the uterine endometrium, real-time RT-PCR was performed using the Applied Biosystems GeneAmp 7300 Sequence Detection System (Applied Biosystems, Foster City, CA) using the SYBR Green method. Complementary DNA was synthesized from 4 μg total RNA isolated from different uterine endometrial tissues, and newly synthesized cDNAs (total volume of 21 μl) were diluted 1:4 with nuclease-free water and then used for PCR. The Power SYBR Green PCR Master Mix (Applied Biosystems) was used for PCR reactions. The final reaction volume of 20 μl included 2 μl of cDNA, 10 μl of 2X Master mix, 2 μl of each primer (100 nM), and 4 μl of dH_2_O. PCR cycle parameters were 50°C for 2 min and 95°C for 15 min followed by 40 cycles of 95°C for 30 s, 60°C for 30 s and 72°C for 30 s. Sequences of primer pairs for PCR reactions and product sizes are listed in Table 1. The results were reported as the relative expression to the level on D12 of pregnancy after normalization of the transcript amount to the two endogenous control genes, porcine ribosomal protein L7 (*RPL7*) and ubiquitin B (*UBB*), by the 2^-ΔΔCT^ method [31]. We performed the Pearson correlation test in order to technically validate the WCGNA results.

**Table 1:** Primer sequences used for real-time RT-PCR.

Gene	GenBank Accession Number	Primer Sequences	Product Sizes (bp)	Annealing Temperature
SLPI	NM_213870	F:5'- ACT GGC TGT CTG TCT TGC AGT GAT T-3'	104	60°C
		R: 5'-TGC TAT CAC GAA CCC AGT TAA GGT G-3'		
CCL28	NM_001024695	F:5'-GAT GTG CCC CTT TAC TGT TCC TCT T-3'	137	60°C
		R:5'-CAG AAG AAT CTG TGT CAG CCC TCA T-3'		
CST6	AY610298	F:5'-CTA CTA CTT CCG CGA CAC CA-3'	205	60°C
		R:5'-GGG AAC CAC AAG GAT CTC AA-3'		
MPZL2	CF787898	F:5'-AAC AGA TGG CCT GAT TGA TGT TCC-3'	110	60°C
		R:5'-ACC CAT GAA TAT GTA ACC AAG ACA CAA-3'		
NFIB	AY609566	F:5'-TTC GGA GAG GGA TAA AAG TCT CCT G-3'	115	60°C
		R:5'-GTG TCA ATC TTC AGA GGT CGC TGT C-3'		
RANBP17	EW220253	F:5'-CTG TCT CGG TTC TTG ACA GAA AGG T-3'	120	60°C
		R:5'-TGA GCT GAG GGC AAG TCT GAT AAA C-3'		
SAL1	NM_213814	F:5'-GCT GAC TCT AGC CTC TTC CCA CAA G-3'	110	60°C
		R:5'-CGT CTG AGG CCA AAA GAA TGG AAT A-3'		
SPP1	EF633681	F:5'-CTC ATT GCT CCC ATC ATA GGT CTT G-3'	111	60°C
		R:5'-CAA GAG AAG GAC AGT CAG GAG ACG A-3'		
SUCLA2	AF061996	F:5'-CAA TGT AGT TGA GAT TTG CCT TCG C-3'	149	60°C
		R:5'-TTC AGA TGG AGC TGT GCT GTG TAT G-3'		
UABP2	NM_213845	F:5'-GAC CTT CCA AAA GTC CTT GTC CTT G-3'	141	60°C
		R:5'-GAC ATA TTC ACT ACC AAG GCC GTC A-3'		
UBB	EF688559	F:5'- AACAGTTCAGTAGTTATGAGCCAGA-3'	65	60°C
		R:5'- AGATGTTCTCAAACGCTTCG-3'		
RPL7	NM_001113217	F:5'-AAG CCA AGC ACT ATC ACA AG-3'	172	60°C
		R:5'-TGC AAC ACC TTT CTG ACC TT-3'		

## Results

### Identification of DEGs in different stages of pregnancy in the uterine endometrium

**Fig 1:**
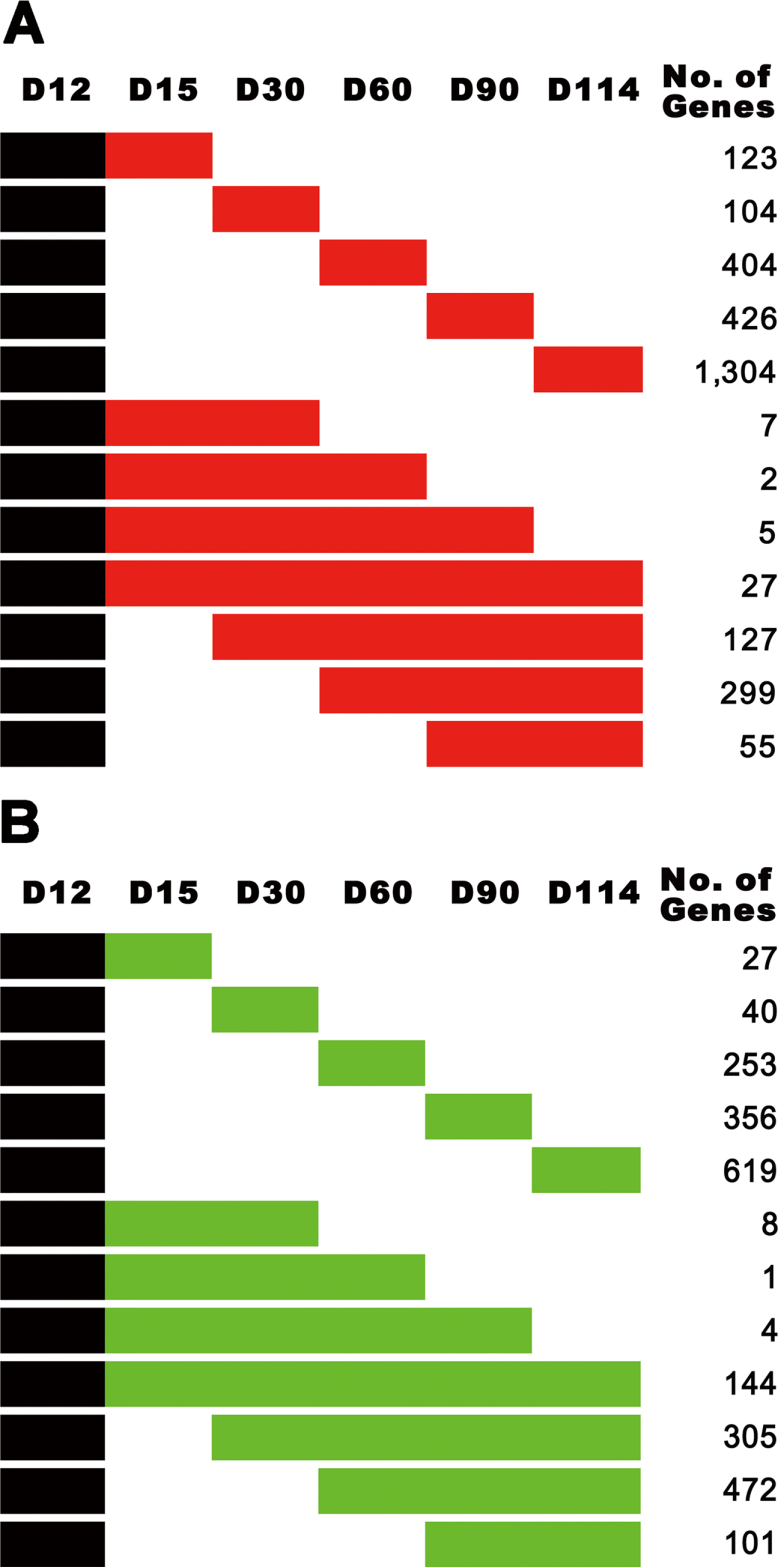
Differentially expressed genes (DEGs) at different stages of gestation compared to genes expressed on day (D) 12 of pregnancy in the uterine endometrium in pigs. (A) Up-regulated genes specifically observed on D15, D30, D60, D90, D114, or stages over more than two different days compared to genes expressed on D12 of pregnancy were re-arranged according to their expression patterns in red. (B) Down-regulated genes specifically observed on D15, D30, D60, D90, D114, or stages over more than two different days compared to genes expressed on D12 of pregnancy were re-arranged according to their expression patterns in green.

We analyzed gene expression profiles from microarray data consisting of 18 endometrial samples (three per each of the 6 selected days of pregnancy) to detect DEGs during pregnancy. For accurate estimation of DEGs, we performed probe and scale normalization using RMA and quantile normalization, respectively. Then, DEGs were detected by comparing genes expressed in the endometrium on D12 of pregnancy with those on D15, D30, D60, D90 and D114 of pregnancy using the LIMMA R package. As a result, we identified DEGs (FDR adjusted P < 0.05) as shown in Table 2. Interestingly, we observed that the number of DEGs during the late stage of pregnancy tended to increase when genes expressed on D12 of pregnancy were compared with genes expressed on D15, D30, D60, D90, and D114 of pregnancy: 256, 449, 1,620, 1,467 and 2,146 genes were significantly up-regulated, and 248, 636, 1,892, 1,947, and 1,884 genes were significantly down-regulated, respectively. Furthermore, we visualized the expression pattern of DEGs during pregnancy, as shown in Fig 1A and 1B, and rearranged all of the up- or down-regulated DEGs by day of pregnancy and their identity (Table 2). Interestingly, many DEGs did not overlap with different stage groups, which suggests that detected DEGs are very stage-specific. In DEG analysis, we observed a total of 6,991 genes without overlapped genes in different stage groups. More detailed information regarding these DEGs is listed in S1 and S2 Tables.

**Table 2:** Number of differentially expressed genes at different stage of gestation compared to the genes expressed on day (D) 12 of pregnancy (P) in the uterine endometrium in pigs.

Comparison	Genes Up-regulated	Genes Down-regulated
D15P / D12P	256	248
D30P / D12P	449	636
D60P / D12P	1,620	1,892
D90P / D12P	1,467	1,947
D114P / D12P	2,146	1,884

### Clustering analysis to determine the global expression pattern of genes during pregnancy

**Fig 2:**
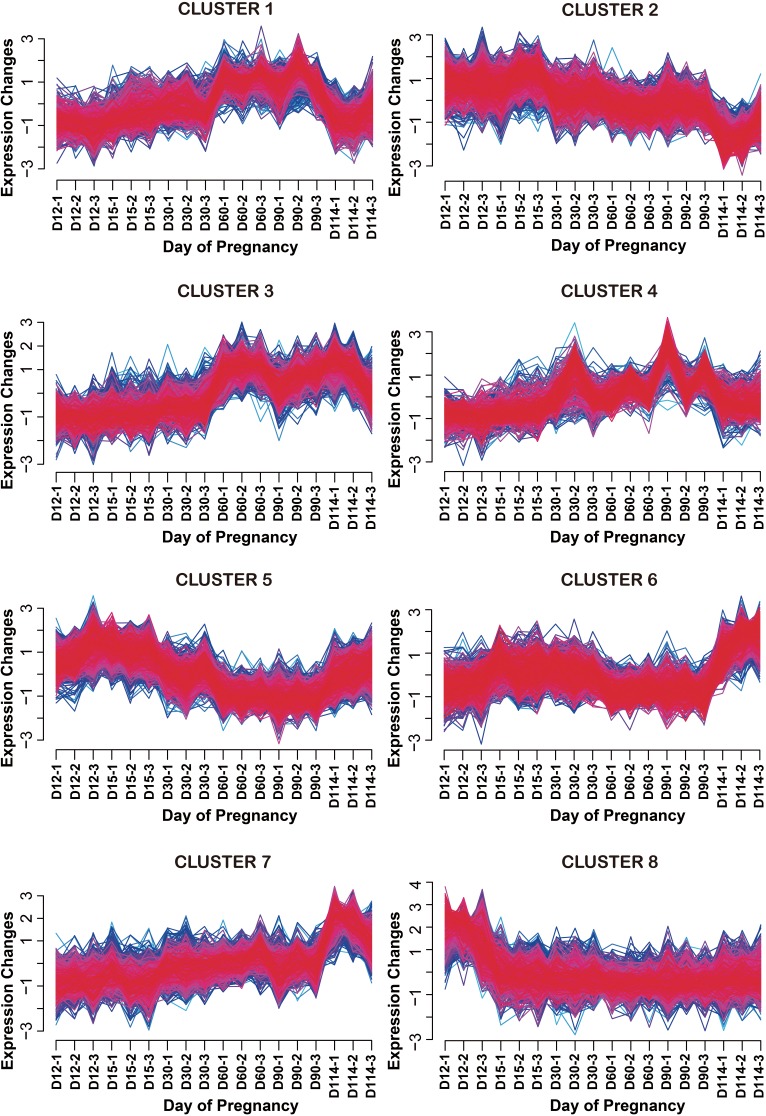
Clustering analysis of differentially expressed genes in the uterine endometrium during pregnancy in pigs. Clustering analysis was performed using the Mfuzz package and dendrograms of each cluster are depicted. The fuzzy c-means (FCM) clustering algorithm, which is a part of the package, uses a soft partitioning clustering method that requires two main parameters (*c* = number of clusters, *m* = fuzzification parameter). In this analysis, the optimal values of *c* and *m* were derived by the iterative refinement procedure. Through this optimization, two parameters were calculated as *c* = 8 and *m* = 1.25. As a result, eight clusters were obtained. Yellow or green lines correspond to genes with a low membership value; red and purple lines correspond to genes with a high membership value. Most genes included in this figure showed a high membership value.

Next, to distinguish the detected DEGs defined above in the endometrium throughout all stages of pregnancy, we conducted clustering analysis using FCM clustering implemented in the mFuzz R package. From the analysis, the optimal number of clusters with a similar expression pattern during pregnancy was calculated to be 8 (Fig 2). Each cluster included 523, 1,059, 831, 820, 1202, 737, 926 and 893 DEGs, respectively. More detailed clustering results with DEGs are shown in S3 Table. In cluster 1, expression of genes increased over the gestation stage and then declined at term. Genes in this cluster were expressed at high levels on D60 to D90 of pregnancy. The representative DEGs included in cluster 1 were *ACSL1*, *ACSL4*, *CYP19A1*, *ICAM1*, *IGFBP1*, *IGFBP5*, *IL10*, *LIF*, *SLPI*, *SPP1* and *UFBP*. On the other hand, cluster 2 tended to be expressed in a gradually decreasing manner throughout gestation, and included *ATP1A1*, *CD14*, *CDKN1B*, *FGFR2*, *GSTA2*, *NCOA1*, *NCOA2*, and *STC1*. In addition, expression of genes in cluster 5 tended to decrease from D60 to D90 of pregnancy. Genes in this cluster included *ATP1B1*, *CTGF*, *ESR1*, *ITGB8*, *OAS1*, *OGT*, and *PGR*. Expression of genes in cluster 7 tended to increase gradually from D12 to D90 of pregnancy and then increase again dramatically at term. *ATP4B*, *CTSL2*, *ESD*, *FBP*, *MMP3*, *NLN*, *PLAT*, and *TIMP1* were included in this cluster. Expression of genes in cluster 8 was high on D12 of pregnancy, and remained low throughout the rest of the pregnancy. Genes included in this cluster were *ALCAM*, *CCL28*, *LPAR3*, *EGF*, *FGF7*, *HIF1A*, *IGF1*, *IL10RB*, *OCLN*, *SAL1* and *SULT1E1*.

### Functional annotation clustering analysis of the DEGs in each cluster to determine biological function during pregnancy

Having determined the global gene expression pattern of DEGs in the uterine endometrium during pregnancy and having identified groups of genes showing a similar expression pattern, we next sought to determine the function of genes in each cluster. To answer this question, we used DAVID functional annotation clustering analysis with annotated gene symbols. We used the highest stringency cut-off criteria for reducing false positives, and obtained enriched functional annotation groups composed of significant gene ontology (GO) terms or biological features in 8 clusters (Table 3 and S4 Table).

**Table 3:** List of enriched functional annotation groups in 8 clusters by functional annotation clustering analysis.

Cluster	Description for Functional Annotation Groups	Enrichment Score*	No of Genes
1	1. Blood vessel development/morphogenesis	6.211	24
	2. Ion/cellular chemical homeostasis	4.421	27
	3. Positive regulation of protein modification/positive regulation of phosphate (phosphorous) metabolic process	4.26	14
	4. Positive regulation of (cellular) proteinmetabolic process	3.464	18
	5. Regulation of apoptosis/programmed cell death	3.354	25
	6. Regulation of axonogenesis/neuron differentiation	2.015	8
	7. Negative regulation of axonogenesis/neurogenesis	1.515	5
	8. Negative regulation of apoptosis/programmed cell death	1.486	17
	9. Gonad development/ovulation cycle process	1.37	7
	10. Mesenchymal cell differentiation/development	1.347	5
2	1. Negative regulation of cellular biosynthetic process/cellular metabolic process	4.926	61
	2. Negative regulation of transcription/RNA metabolic process	4.269	45
	3. Neuron projection development/cell morphogenesis	2.903	30
	4. Regulation of biosynthetic process/transcription	1.92	161
	5. Blood vessel development/morphogenesis	1.532	21
	6. Cellular protein catabolic process/proteolysis	1.53	54
	7. Positive regulation of cellular metabolic process/biosynthetic process/transcription	1.456	59
	8. Mesenchymal cell differentiation/development	1.446	7
3	1. Complement activation/B cell mediated immunity	1.6	6
	2. Positive regulation of protein modification/phosphate (phosphorous) metabolic process	1.464	13
	3. Negative regulation of apoptosis/programmed cell death	1.39	21
4	1. Nucleobase, nucleoside, nucleotide and nucleic acid biosynthetic process/nucleoside phosphate metabolic process	3.17	21
	2. Proton transport/ATP synthesis coupled proton Transport/ion transmembrane transport	3.156	10
	3. Positive regulation of ion transport/regulation of metal ion transport	2.54	10
	4. Cellular response to starvation/nutrient levels	1.873	6
	5. Negative regulation of apoptosis/programmed cell death	1.627	22
5	1. RNA processing/RNAsplicing	22.144	76
	2. Regulation of gene expression/ macromolecule biosynthetic process	5.364	221
	3. Nucleic acid transport/mRNA transport	2.751	15
	4. Negative regulation of nucleobase, nucleoside, nucleotide and nucleic acid metabolic process/ macromolecule metabolic expression/transcription process/gene	2.132	58
	5. Cellular glucan metabolic process/polysaccharide metabolic process/energy reserve metabolic process	2.084	8
6	1. M phase/mitosis	3.506	28
	2. Nucleic acid transport/mRNA transport	3.397	13
	3. tRNA aminoacylation for protein translation/cellular amine metabolic process	1.887	12
	4. Modification-dependent macromolecule catabolic process/protein catabolic process	1.656	35
7	1. Protein amino acid glycosylation/glycoprotein biosynthetic process	2.882	16
	2. Modification-dependent macromolecule catabolic process/protein catabolic process	1.478	37
8	1. Blood vessel development/differentiation	2.862	21
	2. Neuron projection development/cell morphogenesis	2.222	23
	3. Positive regulation of cell migration/cell motion	1.723	11

*; Functional annotation groups resulted from functional annotation clustering analysis using BP terms were ordered by their mean enrichment score. And, it was considered as significant functional groups that were over 1.3 of enrichment score. Gene symbols used in functional annotation clustering analysis were human orthologues corresponding to probe identifications of Porcine Genome Array.

In cluster 1 where gene expression increased over gestation and then declined at term, annotation groups, blood vessel development/morphogenesis, ion/cellular chemical homeostasis, and regulation-related terms were observed. In cluster 2 where gene expression decreased over gestation from early to term pregnancy, annotation groups included negative regulation of cellular biosynthetic/cellular metabolic processes and cellular protein catabolic /proteolysis processes. In cluster 5 where gene expression decreased on D60 to D90 of pregnancy, annotation groups included RNA processing/splicing, regulation of gene expression/macromolecule biosynthetic processes and cellular glucan/polysaccharide/energy reserve metabolic processes. In cluster 8 where gene expression was highest on D12 of pregnancy and remained low thereafter, annotation groups included blood vessel development/differentiation and neuronal projection development/cell morphogenesis.

### Weighted gene co-expression network analysis (WGCNA) to determine groups of closely interrelated genes

**Fig 3:**
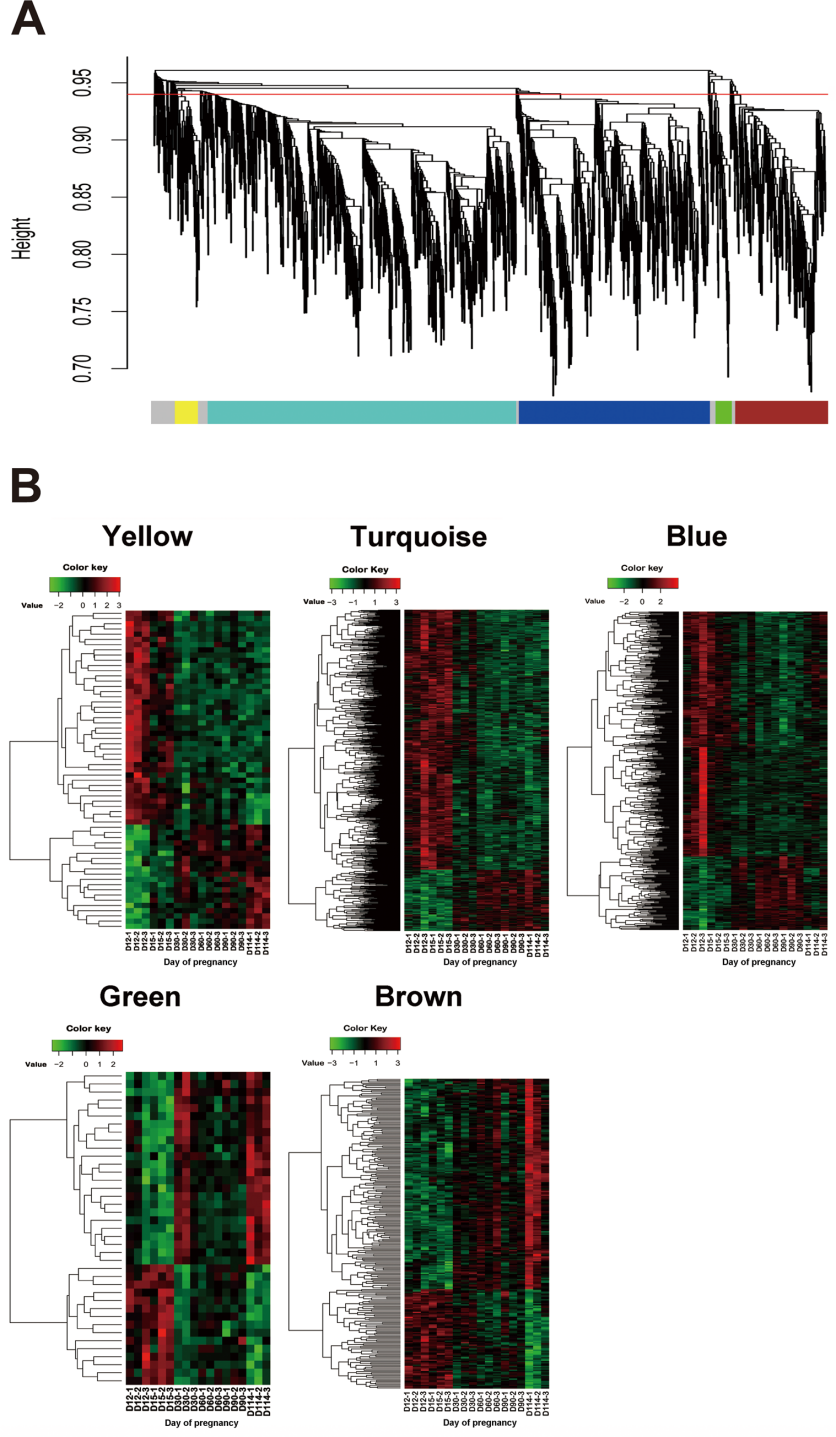
Network analysis of gene expression in the porcine uterine endometrium during pregnancy to identify distinct modules of weighted coexpressed genes. (A) Dendrograms produced by average linkage hierarchical clustering of 24,123 genes based on topology overlap. The red line indicates the height at which the tree was cut (0.94, red line) to define the module. Different modules were assigned colors as indicated in the horizontal bar beneath the dendrogram. (B) Heatmap images of all genes involved in 5 network modules. Columns represent probe identifications and putative annotations, and rows represent day of pregnancy. Red indicates high levels of expression and green indicates low levels of expression.

Next, further efforts were made to determine the interrelationship among genes expressed in the uterine endometrium during pregnancy. We applied WGCNA and identified groups (modules) of closely interrelated genes that had similar patterns of connection strengths to other genes, or high topological overlap [32]. Module detection and characterization were performed using customized R software functions, and gene co-expression modules were identified by average linkage hierarchical clustering with a topological overlap matrix (TOM). As a result, 5 modules in different colors corresponding to branches of the dendrogram as visualized in the TOM plot were obtained by cutting the tree of the dendrogram at the height of 0.94 (Fig 3A). For each module, a heatmap was produced with rows corresponding to genes and columns corresponding to samples ordered by pregnancy stage (Fig 3B). Modules in yellow, turquoise, blue, green, and brown colors included 61, 816, 506, 39, and 246 genes, respectively. A list of genes in each module is provided in S5 Table. Interestingly, heatmaps for 5 modules showed a common pattern of gene expression during pregnancy; genes up-regulated on D12 and D15 of pregnancy were down-regulated after D30 of pregnancy, and genes down-regulated on D12 and D15 of pregnancy were up-regulated after D30 of pregnancy.

We evaluated the intramodular connectivity of genes to each other to identify hub genes that could be markers for specific physiological functions in the uterus. In this network analysis study, hub genes were defined as highly connected genes (> 10 connected nodes). Twenty-five genes that had a connectivity of absolute scaled *K* value greater than 0.9 in each module were selected as hub genes (Table 4). In the yellow module, *SOX13* and *MRPL2* showed the highest connectivity to other genes positively and negatively, respectively. *HNRNPA2B1* and *REXO2* showed the highest connectivity to other genes positively and negatively, respectively, in the turquoise module. In the blue module, *ALPPL2*, *RANBP17* and *NF1B* had the highest connectivity to other genes positively, and *SPP1* and *CST6* had the highest connectivity to other genes negatively. In the green module, *GINS1*, *CDC2* and *KPNA2* were highly connected to genes positively, and *NOLA2* had the highest connectivity to other genes negatively. *MYD88* showed the highest connectivity to other genes positively, and *ATP1A1* showed the highest connectivity to other genes negatively in the brown module.

**Table 4:** List of hub genes that have high connectivity in each module from weighted gene co-expression network analysis.

Module	Probe Identification	Scaled positive K	Scaled negative K	Gene Symbol	Gene Title
Yellow	Ssc.12263.1.A1_at	1	0.4804	SOX13	SRY (sex determining region Y)-box 13
	Ssc.5394.1.S1_at	0.9904	0.4023	CEP290	Centrosomal protein 290kDa
	Ssc.2361.1.A1_at	0.9504	0.499	PPP1R3D	Protein phosphatase 1, regulatory subunit 3D
	Ssc.21783.2.S1_a_at	0.3689	1	MRPL2	Mitochondrial ribosomal protein L2
Turquoise	Ssc.27885.1.S2_at	1	0.1923	HNRNPA2B1	Heterogeneous nuclear ribonucleoprotein A2/B1
	Ssc.30052.1.A1_at	0.9191	0.192	ZNF613	Zinc finger protein 613
	Ssc.9133.1.A1_at	0.2016	1	REXO2	REX2, RNA exonuclease 2 homolog (S. Cerevisiae)
	Ssc.18377.3.S1_a_at	0.1898	0.9515	P2RX4	Purinergic receptor P2X, ligand-gated ion channel, 4
	Ssc.18377.1.S1_at	0.1915	0.9225	P2RX4	Purinergic receptor P2X, ligand-gated ion channel, 4
Blue	Ssc.9533.1.A1_at	1	0.2562	ALPPL2	Alkaline phosphatase,placental-like 2
	Ssc.4154.1.A1_at	0.9672	0.268	RANBP17	Ran Binding Protein 17
	Ssc.28951.1.S1_at	0.9434	0.3428	NFIB	Nuclear factor I/B
	Ssc.12664.2.S1_at	0.9424	0.1939	SLCO3A1	Solute carrier organic anion transporter family, member 3A1
	Ssc.30327.1.A1_at	0.9054	0.2323	TEX9	Testis expressed 9
	Ssc.101.1.S1_at	0.1737	1	SPP1	Secreted phosphoprotein 1
	Ssc.9061.1.A1_at	0.2551	0.9307	CST6	Cystatin E/M
Green	Ssc.5401.1.S1_at	1	0.5092	GINS1	GINS complex subunit 1 (Psf1 homolog)
	Ssc.873.1.S1_at	0.9458	0.4352	CDC2	Cell division cycle 2, G1 to S and G2 to M
	Ssc.11668.1.A1_at	0.9002	0.5182	KPNA2	Karyopherin alpha 2 (rag cohort 1, importin alpha 1)
	Ssc.20730.1.S1_at	0.3122	1	NOLA2	NHP2 ribonucleoprotein homolog (Yeast)
	Ssc.2505.2.S1_at	0.3294	0.9861	AKAP11	A kinase (PRKA) anchor protein 11
Brown	Ssc.23503.1.S1_at	1	0.4688	MYD88	Myeloid differentiation primary response gene (88)
	Ssc.11046.2.S1_at	0.9736	0.4039	SEC22L1	SEC22 vesicle trafficking protein homolog B (S. cerevisiae) (gene/pseudogene)
	Ssc.5789.2.S1_at	0.967	0.4244	SEC22L1	SEC22 vesicle trafficking protein homolog B (S. cerevisiae) (gene/pseudogene)
	Ssc.800.1.S1_at	0.3346	1	ATP1A1	ATPase, Na+/K+ transporting, alpha 1 polypeptide

### Real-time RT-PCR analysis to validate detected hub genes and correlated genes

To demonstrate the correlation of hub genes determined in WGCNA and confirm clustering analysis of DEGs, we conducted real-time RT-PCR for selected genes. We chose *RANBP17* and *SPP1* in the blue module in WGCNA because the blue module included a large number of genes, and *RANBP17* and *SPP1* showed the highest absolute connectivity to other genes in WGCNA. In addition, some genes correlated with hub genes were additionally selected for real-time RT-PCR. Pearson correlation was calculated between hub genes *RANBP17* and *SPP1* and the other genes, as shown in S6 and S7 Tables. Genes positively correlated with *RANBP17* included *SAL1*, *CCL28*, *GALT* and *NF1B3*, and negatively correlated genes included *SPP1*, *SLPI*, *MPZL2*, *UFBP* and *CST6* (S6 Table). Genes positively correlated with *SPP1* included *SLPI*, *MPZL2*, *UFBP*, *CST6* and *SUCLA2*, and negatively correlated genes included *RANBP17*, *SAL1* and *CCL28* (S7 Table).

**Fig 4:**
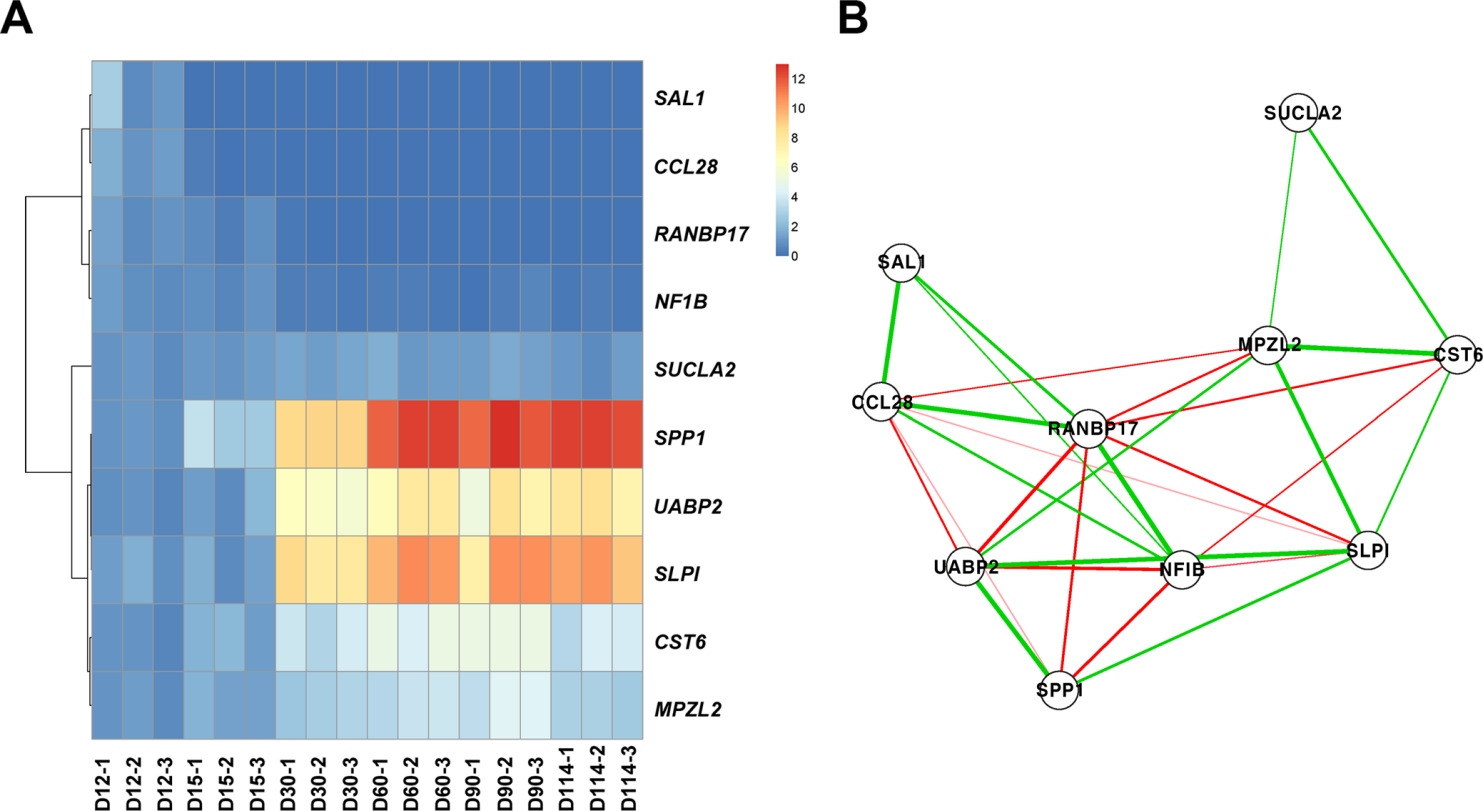
Real-time RT-PCR analysis to technically validate hub genes and correlated genes. (A) Heatmap with hierarchical clustering using 2^-ΔΔCT^ values of 10 genes in real-time RT-PCR. (B) Correlation-based network plot. The edges represent significant correlation (FDR adjusted P-values < 0.05 in Pearson correlation test), and green and red colors represent positive and negative correlation, respectively.

As shown in Fig 4, a heatmap and correlation-based network plot revealed the relationships among the hub genes *RANBP17* and *SPP1* and their correlated genes *SAL1*, *NF1B*, *SLPI*, *MPZL2*, *UFBP*, *CST6*, *CCL28*, and *SUCLA2*. The general expression pattern of these genes in real-time RT-PCR matched the expression pattern of genes in each cluster where those genes were grouped, confirming the accuracy of the microarray analysis. In Fig 4A, expression of most validated genes dramatically changed after D15 of pregnancy except *SAL1* and *CCL28*, the expression of which changed after D12 of pregnancy. We also performed network analysis in real-time RT-PCR in order to identify their relationship. The correlation-based network plot showed these relationships and the Pearson correlation test was used to determine significance. As shown in Fig 4B, significant correlation (FDR adjusted *P*-value < 0.05) and their direction (positive or negative) are visualized as a network plot. As a result, all genes were significantly correlated each other. As expected, *RANBP17* and *SPP1* were observed as hub genes in real-time RT-PCR. *NFIB*, *SAL1*, and *CCL28* were highly positively correlated with *RNABP17* and *SPP1*, *UABP2*, *SLP1*, *MPZL2*, and *CTS6* were highly negatively correlated with *RNABP17*. These results confirmed the hub genes detected in WGCNA.

## Discussion

The uterine endometrium plays an essential role in the establishment and maintenance of pregnancy and undergoes morphological and functional changes during pregnancy in mammals. To support pregnancy, expression of uterine endometrial genes alters dynamically depending on the stage of gestation and is affected by hormones and cytokines of ovarian, conceptus, chorionic and other origins. Although many studies have investigated the expression and function of endometrial genes in pigs, the genes expressed in the uterine endometrium and the cellular and molecular function of those gene products during pregnancy are not completely understood. In this study, we analyzed the global expression patterns of genes in the endometrium from the early to the late stage of pregnancy using a microarray technique in pigs. As a result, we determined the following: 1) genes are expressed differentially during the different stages of pregnancy; 2) groups of genes show similar expression patterns during pregnancy. We also evaluated the functional annotation of grouped genes and the correlation of genes expressed in the endometrium during pregnancy and found that hub genes closely correlated with other genes. To our knowledge, this is the first report investigating the global expression patterns of uterine endometrial genes throughout gestation in pigs.

Expression of endometrial genes is regulated by many factors and changes dynamically during pregnancy. In this study we identified DEGs by comparing expression levels of endometrial genes on D15, D30, D60, D90 and D114 of pregnancy with those on D12 of pregnancy, which is when maternal recognition of pregnancy occurs and embryo implantation begins in pigs [1]. Using clustering analysis we grouped those DEGs into 8 clusters based on the overall expression pattern throughout pregnancy, and the genes in each cluster and their functional annotation groups represented unique physiological functions during specific periods of pregnancy. Expression of DEGs in cluster 1 increased throughout gestation and declined at term. Genes following this pattern included *ACSL1*, *ACSL4*, *CYP19A1*, *ICAM1*, *IGFBP1*, *IGFBP5*, *IL10*, *LIF*, *SLPI*, *SPP1* and *UFBP*. Some genes such as *SLPI*, *SPP1* and *UFBP* have been previously investigated [33–35], and our results coincide with the expression pattern of those genes in previous studies. Because genes in this cluster were highly expressed during mid-to-late pregnancy when placental growth and fetal development reach the maximum levels [4, 5], it is likely that genes in this cluster are involved in support of fetal/placental development and endometrial remodeling. Indeed, functional annotation clustering analysis showed that the gene function of this group includes blood vessel development/morphogenesis, ion/cellular chemical homeostasis, positive regulation of protein modification, and mesenchymal cell differentiation. Genes in cluster 5 showed a pattern of decreased expression on D60 to D90 during pregnancy and included *ATP1B1*, *CTGF*, *ESR1*, *ITGB8*, *OAS1*, *OGT*, and *PGR*. Functional annotation groups for the genes in this cluster include RNA processing/splicing, regulation of gene expression/macromolecule biosynthetic process and cellular glucan/polysaccharide/energy reserve metabolic process. Genes in cluster 7 showed a pattern of gradually increasing expression from D12 to D90 of pregnancy and had the highest levels at term. These genes include *ATP4B*, *CTSL2*, *ESD*, *FBP*, *MMP3*, *NLN*, *PLAT*, and *TIMP1*. Functional annotation groups in this cluster include protein amino acid glycosylation/glycoprotein biosynthetic processes and modification-dependent macromolecule catabolic/protein catabolic processes, which suggests that remarkable endometrial remodeling occurs in the preparation for parturition at term. Genes in cluster 8 showed highest expression on D12 of pregnancy and decreased thereafter during pregnancy, and include *ALCAM*, *CCL28*, *EGF*, *FGF7*, *HIF1A*, *IGF1*, *IL10RB*, *LPAR3*, *OCLN*, *SAL1* and *SULT1E1*. Functional annotation groups include blood vessel development/differentiation and neuronal projection development/cell morphogenesis. The highest levels of expression on D12 of pregnancy for the genes such as *FGF7* [36], *LPAR3* [37], *SAL1* [38], and *SULT1E1* [39, 40] have already been reported, confirming the accuracy of microarray analysis in this study.

In WGCNA to determine groups of closely interrelated genes expressed in the uterine endometrium during pregnancy, we identified 5 groups (modules) of closely interrelated genes that had similar patterns of connection strengths to other genes, or high topological overlap. Since hub genes are known to play a critical role in the genetic interaction network, we assumed that these genes function in expression and regulation of other genes in the same module in the uterine endometrium during pregnancy and that they might be markers for predicting specific uterine gene function. Twenty-four hub genes were identified based on connectivity of absolute scaled K values of more than 0.9 in each module. Among them, *SPP1*, *CST6*, and *RANBP17* were detected as hub genes in the blue module. Indeed, many studies have shown the critical roles of these genes in the uterine endometrium.

The importance of *SPP1* in endometrial function during pregnancy is well understood in many species, including humans, rabbits, pigs, and ruminants [41]. SPP1, also called osteopontin and early T-cell activator factor 1, is an acidic glycosylated phosphoprotein found in all body fluids and in a variety of tissues. SPP1 acts on various biological processes such as cell migration, inflammation, activation of B and T cells, and bone formation [41]. In pigs, *SPP1* is expressed in uterine endometrial luminal epithelial cells from early stage of pregnancy and is also expressed in glandular epithelial cells from mid- to late-stage of pregnancy [34]. SPP1 can undergo dramatic post-translational modifications such as phosphorylation, glycosylation, and cleavage, forming different sizes of variants including native 75-kDa, 45- and 25-kDa forms [41]. SPP1, an extracellular matrix (ECM) protein, binds multiple integrins through Arg-Gly-Asp-mediated or alternative integrin recognition sequences to effect cell-cell and cell-ECM adhesion [42]. In pigs, SPP1 binds specific integrins and promotes trophectoderm cell migration and attachment to luminal epithelial cells [43]. These suggest that SPP1 plays a critical role in cell-cell and cell-ECM adhesion at the maternal-conceptus interface, especially in pigs, which form a true non-invasive epitheliochorial placentation. Furthermore, several studies have shown that the *SPP1* gene is associated with litter size and prenatal survival. The porcine *SPP1* gene is included in quantitative trait loci (QTL) associated with reproductive performance such as ovulation rate, litter size, and prenatal survival on chromosome 8 [44]. A polymorphism has been detected in an intron region of the *SPP1* gene [45, 46], and is associated with birth body weight, growth rate, and carcass traits in the Landrace X Jeju (Korea) black pig F_2_ population [46]. In addition, it has been shown that expression of SPP1 is higher in the uterine endometrium of the Meishan pig, a highly prolific pig breed, than in the hyperprolific Large White breed, suggesting the association of *SPP1* with placental efficiency during pregnancy [47]. Therefore, the finding of *SPP1* as a hub gene in the uterine endometrium during pregnancy in this study and the role of SPP1 in the uterine endometrium during pregnancy and association of the *SPP1* gene with porcine litter size and prenatal survival indicates that SPP1 is a critical factor for regulating uterine-conceptus interactions and maintaining a successful pregnancy.

In this study, *CST6* was also identified as a hub gene in the uterine endometrium during pregnancy. CST6 (cystatin 6 or cystatin E/M) is an inhibitor of cathepsins (CTSs), including CTSB, CTSL, and legumain (LGMN) [48], which are lysosomal cysteine proteases acting on degradation of extracellular matrix molecules and activation of intracellular pre-proteins [49]. Expression of CTSs and CSTs in the uterine endometrium has been shown in many species including rodents, humans, ruminants and pigs [50–52], and it has been suggested that CTSs and CSTs play important roles in endometrial remodeling in the uterine endometrium during the reproductive cycle and pregnancy [52]. In pigs, CTSB, CTSL1, and LGMN are expressed by endometrial luminal epithelial and chorionic epithelial cells at the maternal-conceptus interface during pregnancy and endometrial expression of CTSB and CTSL1 is induced by progesterone [51, 53], suggesting that their action is required to remodel uterine endometrial and placental tissues and facilitate transplacental transport of nutrients. CST6 is expressed in the uterine endometrium during the estrous cycle and in pregnancy, and also in the chorionic epithelia of the placental membrane with increasing levels during late pregnancy [53], suggesting that cell type-specific expression and function of CST6 is critical in regulation of CTS action for appropriate maternal-fetal interactions.

The result of this study showed that *RANBP17* is a hub gene in the endometrium during pregnancy in pigs. RANBP17 is a member of the importin-β superfamily of nuclear transporter receptors that are involved in the nucleocytoplasmic shuttling of many cargo proteins and interact directly with RanGTP to modulate the compartment-specific binding of their substrate [54]. Importin-βs play essential roles in diverse cellular processes such as gene expression, signal transduction, oncogenesis, cell division, and nuclear envelope assembly [55]. Expression of *RANBP17* has been detected in many human and mouse tissues, including brain, heart, lung, pancreas, placenta, and testis, with the highest levels of expression in testis [54], and levels of *RANBP17* mRNAs increases with ischemic and dilated cardiomyopathy [56]. Although the specific function of RANBP17 has not been well understood, it has been shown that RANBP17 interacts with a basic helix-loop-helix transcription factor, E12, and increases transcriptional activity of E12, suggesting that E12 may be a cargo protein for RANBP17 [57]. Even though expression and function of *RANBP17* in the female reproductive tract have not been determined, our result suggests that RANBP17 may play a critical role in the shuttling and regulation of protein cargoes such as transcription factors in the uterine endometrium during pregnancy.

Using real-time RT-PCR analysis to determine endometrial expression of hub genes, *RANBP17* and *SPP1* and their correlated genes and to demonstrate the correlation of hub genes determined in WGCNA, we found that the genes positively or negatively correlated with hub genes showed the same or opposite patterns of expression to each other in the uterine endometrium during pregnancy. These results confirmed the correlation among the hub genes. Furthermore, general expression patterns of the hub genes and their correlated genes matched the patterns determined by clustering analysis of DEGs.

In conclusion, our study revealed the complex network characteristics involved in uterine endometrial gene expression during pregnancy stages. Our findings suggest that the diverse patterns of stage-specific gene expression and network connections observed during pregnancy play critical roles in endometrial remodeling and in placental and fetal development in pigs. Further study of the hub genes identified in this study would provide insight into the interrelationship of genes expressed in the uterine endometrium and their endometrial function during pregnancy in pigs.

## Supporting Information

S1 Table List of up-regulated genes compared with D12P. (XLSX)

S2 Table List of down-regulated genes compared with D12P. (XLSX)

S3 Table List of genes in each cluster. (XLSX)

S4 Table Functional annotation clustering analysis of 8 clusters. (XLSX)

S5 Table Annotation of each network module. (XLSX)

S6 Table Genes correlated with *RANBP17*. (DOCX)

S7 Table Genes correlated with *SPP1*. (DOCX)
