# Supplementary material for: Analysis of Stage-Specific Gene Expression Profiles in the Uterine Endometrium during Pregnancy in Pigs
Source: PLoS One. 2015 Nov 18;10(11):e0143436. doi: 10.1371/journal.pone.0143436 (PMC4651506; doi:10.1371/journal.pone.0143436)
Supplement: S6 Table — (DOCX) [file pone.0143436.s006.docx]

**Supplementary Table 6a.** List of genes highly correlated with *RANBP17* positively or negatively in weighted gene co-expression network analysis.

**Probe Identification r^2^ Gene Symbol** **Gene Title**Ssc.101.1.S1_at -0.9520 *SPP1*  Secreted phosphoprotein 1

Ssc.6080.1.S1_at -0.9369 *SLPI* Secretory leukocyte peptidase inhibitor

Ssc.22310.1.S1_at -0.9312 *MPZL2* Myelin protein zero-like 2

Ssc.22310.2.A1_at -0.9049 *MPZL2* Myelin protein zero-like 2

Ssc.12341.1.S1_at -0.8910 *UFBP* Uteroferrin-associated protein

Ssc.9061.1.A1_at -0.8774 *CST6* Cystatin E/M

Ssc.18072.1.A1_at -0.8730 *OSMR* Oncostatin M receptor

Ssc.18314.1.S1_at -0.8717 *KDSR* 3-ketodihydrosphingosine reductase

Ssc.8305.1.A1_at -0.8687 Null 891995, Sus scrofa cDNA 3-

Ssc.7063.1.A1_at -0.8611 *MMGT1* Membrane magnesium transporter 1

Ssc.809.1.S1_at -0.8577 *IFI30* Interferon, gamma-inducible protein 30

Ssc.4271.1.S1_at -0.8568 Null Sus scrofa mRNA, clone:

VRM10185H10, expressed in ovary

Ssc.7608.1.A1_at -0.8554 *ARL8B* ADP-ribosylation factor-like 8B

Ssc.1049.1.S1_at -0.8540 *COL12A1* Collagen, type XII, alpha 1

Ssc.9351.1.A1_at -0.8446 Null UMC-p4mm3-017-c03,

Sus scrofa cDNA 3-

Ssc.16515.1.A1_at -0.8348 *FOXN2* Forkhead box N2

Ssc.4529.1.S1_at -0.8334 *SLC36A2* Solute carrier family 36 (proton/amino

acid symporter), member 2

Ssc.3285.1.S1_at -0.8266 *ST14* Suppression of tumorigenicity 14

(colon carcinoma)

Ssc.1743.1.S1_at -0.8244 *MAP17* Membrane-associated protein 17

Ssc.14211.1.A1_at -0.8235 Null Sus scrofa mRNA, clone:MLTL10005H08,

expressed in longissimus

Ssc.9533.1.A1_at 0.9846 Null MI-P-AY1-nra-a-06-0-UI.s1,

Sus scrofa cDNA clone

Ssc.3750.1.S1_at 0.9694 *MTCH1* Mitochondrial carrier homolog 1

(C. Elegans)

Ssc.18264.1.S1_at 0.9518 *GLT25D2* Glycosyltransferase 25 domain

containing 2

Ssc.29108.1.S1_at 0.9494 *CBFB* Core-binding factor, beta subunit

Ssc.266.1.S1_at 0.9466 *SAL1*  Salivary lipocalin

Ssc.25689.1.S1_at 0.9462 *ZNF263*  Zinc finger protein 263

Ssc.30498.1.A1_at 0.9421 Null UMC-pd6end2-006-g05,

Sus scrofa cDNA clone

Ssc.29100.1.S1_at 0.9418 *CCL28* Chemokine (C-C motif) ligand 28

Ssc.1639.2.S1_a_at 0.9337 *GALT* Galactose-1-phosphate uridylyltransferase

Ssc.4987.1.A1_at 0.9325 *IFT122* Intraflagellar transport 122 homolog

(Chlamydomonas)

Ssc.6280.1.A1_at 0.9321 Null MI-P-CP1-nzb-h-04-0-UI.s1,

Sus scrofa cDNA clone

Ssc.1639.1.A1_at 0.9310 *GALT* Galactose-1-phosphate uridylyltransferase

Ssc.3046.2.S1_a_at 0.9272 *ARP3* Actin-related protein 3 homolog B (yeast)

Ssc.28951.1.S1_at 0.9265 *NFIB*  Nuclear factor I/B

Ssc.29532.1.A1_at 0.9259 Null UMC-peov3-006-g06,

Sus scrofa cDNA clone

Ssc.27833.1.A1_at 0.9221 *MTMR10* Myotubularin related protein 10

Ssc.12664.2.S1_at 0.9218 *SLCO3A1* Solute carrier organic anion transporter

family, member 3A1

Ssc.16896.1.A1_at 0.9211 Null UNL-P-FN-al-c-11-0-UNL.s1

Sus scrofa cDNA clone

Ssc.12355.1.A1_at 0.9209 Null MI-P-CP0-nvt-d-03-0-UI.s1 Sus scrofa

cDNA clone

Ssc.8805.1.A1_at 0.9200 *NFIB* Nuclear factor I/B
