# Supplementary material for: Analysis of Stage-Specific Gene Expression Profiles in the Uterine Endometrium during Pregnancy in Pigs
Source: PLoS One. 2015 Nov 18;10(11):e0143436. doi: 10.1371/journal.pone.0143436 (PMC4651506; doi:10.1371/journal.pone.0143436)
Supplement: S7 Table — (DOCX) [file pone.0143436.s007.docx]

**Supplementary Table 6b.** List of genes highly correlated with *SPP1* positively or negatively in weighted gene co-expression network analysis.

**Probe Identification r^2^ Gene Symbol** **Gene Title**Ssc.9533.1.A1_at -0.9637 Null MI-P-AY1-nra-a-06-0-UI.s1,

Sus scrofa cDNA clone

Ssc.3750.1.S1_at -0.9577 *MTCH1* Mitochondrial carrier homolog 1

(C. elegans)

Ssc.4154.1.A1_at -0.9520 *RANBP17* RAN binding protein 17

Ssc.29100.1.S1_at -0.9480 *CCL28* Chemokine (C-C motif) ligand 28

Ssc.18264.1.S1_at -0.9468 *GLT25D2* Glycosyltransferase 25 domain

containing 2

Ssc.30552.1.A1_at -0.9304 *STRBP* Spermatid perinuclear RNA binding protein

Ssc.266.1.S1_at -0.9303 *SAL1* Salivary lipocalin

Ssc.25689.1.S1_at -0.9257 *ZNF263* Zinc finger protein 263

Ssc.30498.1.A1_at -0.9198 Null UMC-pd6end2-006-g05,

Sus scrofa cDNA clone

Ssc.1905.1.A1_at -0.9149 Null MI-P-A2-aez-c-02-1-UM.s1,

Sus scrofa cDNA clone

Ssc.12355.1.A1_at -0.9075 Null MI-P-CP0-nvt-d-03-0-UI.s1,

Sus scrofa cDNA clone

Ssc.2478.1.S1_at -0.9003 *PPP1R3*  Protein phosphatase 1, regulatory (inhibitor)

subunit 3B

Ssc.1639.2.S1_a_at -0.8998 *GALT* Galactose-1-phosphate uridylyltransferase

Ssc.12664.2.S1_at -0.8945 *SLCO3A1* Solute carrier organic anion transporter

family, member 3A1

Ssc.26649.1.A1_at -0.8937 Null Pig DNA sequence from clone CH242-

223P6 on chromosome X

Ssc.2860.2.S1_a_at -0.8936 *FAF*2 Fas associated factor family member 2

Ssc.1639.1.A1_at -0.8892 *GALT* Galactose-1-phosphate uridylyltransferase

Ssc.5113.2.A1_at -0.8882 C2orf67 Chromosome 2 open reading frame 67

Ssc.30965.1.A1_at -0.8868 Null UMC-p8mm4-011-c02,

Sus scrofa cDNA clone

Ssc.16896.1.A1_at -0.8867 *ARL6IP1* ADP-ribosylation factor-like 6

interacting protein 1

Ssc.6080.1.S1_at 0.9129 *SLPI*  Secretory leukocyte peptidase inhibitor

Ssc.22310.1.S1_at 0.8898 *MPZL2* Myelin protein zero-like 2

Ssc.17325.1.S1_at 0.8833 *CD24L4*  CD24 molecule-like 4 pseudogene

Ssc.12341.1.S1_at 0.8784 *UABP* Uteroferrin-associated protein

Ssc.22310.2.A1_at 0.8682 *MPZL2* Myelin protein zero-like 2

Ssc.18072.1.A1_at 0.8652 *OSMR* Oncostatin M receptor

Ssc.16515.1.A1_at 0.8621 *FOXN2* Homo sapiens forkhead box N2

Ssc.14211.1.A1_at 0.8457 Null Sus scrofa mRNA, clone:MLTL10005H08,

expressed in longissimus

Ssc.28944.1.S1_at 0.8442 Null 230344, MARC 2PIG Sus scrofa cDNA 5-

Ssc.9061.1.A1_at 0.8319 *CST6* Cystatin E/M

Ssc.4529.1.S1_at 0.8293 *SLC36A2* Solute carrier family 36 (proton/amino acid

symporter), member 2

Ssc.8305.1.A1_at 0.8287 Null 891995, MARC 4PIG Sus scrofa cDNA 3-

Ssc.809.1.S1_at 0.8273 *IFI30* Interferon, gamma-inducible protein 30

Ssc.3285.1.S1_at 0.8194 *ST14* Suppression of tumorigenicity 14

(colon carcinoma)

Ssc.4271.1.S1_at 0.8188 EST 933243, MARC 4PIG Sus scrofa cDNA 3-

Ssc.960.1.S1_at 0.8178 *CANX* Calnexin

Ssc.68.1.A1_at 0.8077 *SUCLA2* Succinate-CoA ligase, ADP-forming,

beta subunit

Ssc.7063.1.A1_at 0.7965 *MMGT1* Membrane magnesium transporter 1

Ssc.1332.1.S1_at 0.7945 *SULT2A1* Sulfotransferase family, cytosolic, 2A,

dehydroepiandrosterone (DHEA)-

preferring, member 1

Ssc.7608.1.A1_at 0.7863 *ARL8B* ADP-ribosylation factor-like 8B
